# Health Care Utilization Databases obtained from health system inform outcome for ruxolitinib treatment in patients with myelofibrosis

**DOI:** 10.1002/hem3.70316

**Published:** 2026-02-24

**Authors:** Barbara Mora, Matteo Franchi, Ludovica Margotto, Olivia Leoni, Daniela D'ippoliti, Emanuela Carloni, Ilaria Cozzi, Enrica Santelli, Fabrizio Gemmi, Claudia Szasz, Margherita Maffioli, Carmelo Gurnari, Enrico Attardi, Daniele Cattaneo, Marta Bortolotti, Nicola Stefano Fracchiolla, Alessandra Iurlo, Giovanni Corrao, Matteo Giovanni Della Porta, Alessandro Maria Vannucchi, Maria Teresa Voso, Paola Guglielmelli, Francesco Passamonti

**Affiliations:** ^1^ Hematology, Fondazione IRCCS Ca' Granda Ospedale Maggiore Policlinico Milan Italy; ^2^ National Center for Healthcare Research and Pharmacoepidemiology University of Milano‐Bicocca Milan Italy; ^3^ Unit of Biostatistics, Epidemiology and Public Health University of Milano‐Bicocca Milan Italy; ^4^ Regional Epidemiological Observatory Lombardy Region Milan Italy; ^5^ Department of Epidemiology Lazio Regional Health Service Rome Italy; ^6^ Quality and Equity Unit Regional Health Agency of Tuscany Florence Italy; ^7^ Hematology, Ospedale di Circolo ASST Sette Laghi Varese Italy; ^8^ Department of Biomedicine and Prevention University of Rome Tor Vergata Rome Italy; ^9^ Comprehensive Cancer Center IRCCS Humanitas Research Hospital Rozzano Italy; ^10^ Department of Biomedical Sciences Humanitas University Pieve Emanuele Italy; ^11^ Centro di Ricerca e Innovazione Malattie Mieloproliferative (CRIMM), AOU Careggi University of Florence Florence Italy; ^12^ Department of Oncology and Hemato‐Oncology University of Milan Milan Italy

## Abstract

Ruxolitinib (RUX) is a JAK1/2 inhibitor widely used in patients with myelofibrosis (MF). Here, we provided real‐world data on 652 intermediate‐2 and high‐risk MF patients receiving RUX, by analyzing electronic Health Care Utilization Databases (HCUD) of all individuals that started RUX in three Italian regions (Lombardy, Lazio, and Tuscany) between October 2014 and December 2017. Over 9 years of observation, the median follow‐up of the cohort was 36.8 months. HCUD of this cohort provided relevant information, (1) contemporary rate of patients on RUX receiving stem cell transplantation: 10.9%; (2) median time to RUX discontinuation: 31.2 months (95% confidence interval [CI]: 26.4–36); (3) transfusions need in the first 6 months of RUX: no red blood cell (RBC) units in 408 (69%), 1–5 in 172 (29%), ≥6 in 14 (2%); (4) events' incidence rate (×100 person‐years) that led to hospital admission: 10.3 for infections, 5.47 for solid tumors, 3.47 for bleeding, 1.56 for thrombosis, and 5.22 for accelerated/blast phase; (5) RUX individual average cost rate in Italy: 30,675 €/year, increasing with worsening *Multisource Comorbidity Score* (MCS). Finally, the median survival was 48 months (95% CI: 43.2–51.6). In a multivariable Cox model, together with patient‐related factors, starting RUX doses below 20 mg every 12 h (BID) were associated with increased mortality (P from 0.007 to <0.001). We report a novel HCUD‐based approach to provide critical healthcare information in the field of MF, based on large populations of patients with documented follow‐up.

## INTRODUCTION

Myelofibrosis (MF) is a *BCR::ABL1*‐negative myeloproliferative neoplasm (MPN) characterized by heterogeneous alterations of the blood counts, bone marrow fibrosis, splenomegaly, constitutional symptoms, and vascular complications.^1^, ^2^ Besides, MF has an inherent tendency to evolve into accelerated phase (AP) and secondary acute myeloid leukemia (also known as blast phase, BP).^1^, ^2^, ^3^, ^4^ MF includes primary MF (PMF), which comprises prefibrotic‐ (pre) and overt‐PMF, and secondary MF (SMF), in case of a previous history of polycythemia vera (PV) or essential thrombocythemia (ET).^1^, ^2^

Median overall survival (OS) of pre‐PMF, overt‐PMF, and SMF patients is estimated to be 14, 7, and 9 years, respectively.^2^ The main causes of mortality are non‐clonal progression of disease and transformation into BP.^2^ Accurate survival estimates are based on prognostic models as the *International Prognostic Scoring System* (IPSS), the *Dynamic IPSS* (DIPSS), or the mutation‐enhanced ones (MIPSS‐70 and variants) for PMF and the *MYelofibrosis SECondary‐Prognostic Model* (MYSEC‐PM) for SMF.^2^ Based on such scores, a patient with an expected survival below 5 years, fit and with a suitable donor, would be a candidate to allogeneic hematopoietic stem cell transplantation (allo‐SCT),^2^, ^5^ in case of a predicted positive post‐SCT outcome.^6^, ^7^, ^8^ Irrespectively of allocation to allo‐SCT, JAK inhibitors (JAKis) are the standard of care for achieving an effective control of disease burden.^2^

Ruxolitinib (RUX) is the first approved JAKi, with more mature data in MF.^2^ RUX showed an undoubted efficacy in reducing splenomegaly and symptoms.^2^ A favorable impact on OS has been reported in post hoc analysis of the registrational trials,^9^, ^10^, ^11^ and in heterogeneous studies of real‐world data (RWD).^12^, ^13^, ^14^, ^15^, ^16^, ^17^ In addition, some clinical findings and molecular profiles on RUX treatment are known to predict response and outcome.^17^, ^18^, ^19^, ^20^ The safety profile of RUX is well defined^2^, ^21^: anemia and thrombocytopenia are frequent and mostly evident in higher‐risk MF, with Grade 3/4 events respectively reported in around 45% and 13% of cases in the registrational trials.^2^ Hematological toxicity is often managed with dose modifications, transfusions, or both.^2^, ^21^ Infectious complications are also a concern, due to JAK1 inhibition.^21^, ^22^ Out of 446 MF subjects (over 80% in the higher‐risk groups) treated with RUX in a real‐world (RW) setting, 28% experienced infectious events (one‐third being Grade 3/4).^22^ As for second primary malignancies (SPM), different reports have underlined just an association between non‐melanoma skin cancers (NMSC) and RUX,^21^, ^23^, ^24^, ^25^ however, in some instances, impacting on survival.^25^ No significant correlations with other SPM have been revealed.^23^, ^24^, ^25^ Across different studies, around half of patients discontinue RUX at 3 years,^26^, ^27^ mostly for progression or intolerance, with a subsequent median survival below 2 years.^26^, ^27^ Preliminary data collected in RW settings have shown a potential survival benefit in switching to other JAKis, such as momelotinib (MMB) or fedratinib.^28^, ^29^

In Italy, RUX has been available since October 2014 for intermediate‐2 (int‐2) and high‐risk (HR) MF patients, and also since January 2018 for intermediate‐1 (int‐1) MF cases and for PV patients after failure of first‐line treatment. High‐cost drugs such as RUX^30^, ^31^ are delivered and monitored by every regional health authority.

In this study, we investigated the outcome of a population‐based cohort including all int‐2 and HR MF patients that started RUX outside a clinical trial between October 2014 and December 2017, by analyzing their electronic Health Care Utilization Databases (HCUD)^32^ over 9 years of observation, accounting for a median follow‐up of 36.8 months. The population included individuals from three Italian regions, Lombardy, Lazio, and Tuscany—accounting for almost 20 million people and involving major cities such as Milan, Rome, and Florence. The information coming from RWD is paramount as it could accelerate clinical research on MPNs with a limited cost, enabling critical information on RUX dose, individual episodes of care, and outcomes in MF patients.^33^, ^34^

## METHODS

### Study design and data sources

This is a population‐based retrospective study on the HCUD of Lombardy, Lazio, and Tuscany regions in Italy. The Italian *National Health Service* (NHS) provides universal and mostly free healthcare services, which are stored in an automated HCUD.^32^ HCUD collect a variety of information, including (a) demographic and administrative data on residents who receive NHS assistance (almost the whole resident population); (b) hospital discharge records providing information on primary reason for admission, coexisting conditions, and procedures performed to inpatients admitted in public and private hospitals and coded according to the *International Classification of Diseases, 9th Revision, Clinical Modification*; (c) drugs dispensed by territorial pharmacies and medicines directly administered in the outpatient setting and day‐hospital coded according to the *Anatomical Therapeutic Chemical* classification system; (d) data on outpatient services, including specialist medical visits, laboratory tests, and diagnostic imaging; and (e) co‐payment exemption database, including exemption for cancer, coded according to the National nomenclature.^32^ Record linkage among databases is allowed through a single identification code (Regional Health Code).^32^

### Study population and covariates

The study cohort included all beneficiaries of the three Regional Health Services (RHS) who started RUX outside a clinical trial between 1 October 2014 and 31 December 2017 and with at least 2 years of recordings in the RHS. Due to the overmentioned HCUD characteristics, we identified these patients as being diagnosed with int‐2 and HR MF because of the RUX label indication in Italy. Subjects that received phlebotomies within 1 year before or after starting RUX were excluded. Alternative JAKis were not accessible in Italy during that period in the clinical practice setting.

Available data at the time of the first RUX prescription were sex, age, RUX initial dose, and the *Multisource Comorbidity Score* (MCS, detailed in Supplementary Methods).

### Outcomes of interest and follow‐up

The primary outcome of interest was survival, defined as the time between the start of RUX therapy and death from any cause or censoring due to patient migration (that means the transfer of a patient to a city outside the region of initial residence), allo‐SCT or end of data availability due to different regional data protection rules (31 December 2023 in Lombardy and Lazio, and 31 July 2021 in Tuscany).

Then we evaluated time to treatment discontinuation (time to TD, TTD), defined as the time between the start of RUX therapy and drug discontinuation for any cause (including death) or censoring due to patient migration or end of data availability.

We also analyzed the number of red blood cell (RBC) units transfused during the first 6 months of treatment, compared to that received at baseline (defined as the period within 3 months before the start of RUX treatment).

The incidence of adverse events (AEs), SPM, AP, and BP that required hospital admission was assessed. AEs included infections, hemorrhagic events (all major), thrombosis, and splenectomy, which were coded in the HCUD as the main reason for hospital admission. AEs occurred within 90 days from the last RUX prescription and before an eventual AP/BP.

Finally, we evaluated the average per capita cumulative healthcare costs sustained by the NHS, from the start of RUX therapy to the earliest date between death, patient migration, or the end of data availability. The costs related to RUX supply were obtained by pharmacy dispensing records, reflecting the actual amount of drug administered to each patient.

### Statistical analyses

Descriptive measures were used to summarize baseline characteristics. OS and TTD were estimated by between‐region summarized Kaplan–Meier (KM) curves (detailed in Supplementary Methods). A multivariable Cox regression model was used to identify risk factors associated with mortality: sex, age classes (<70 vs. 70–79 vs. ≥80 years), MCS categories, year of start of RUX therapy and its initial dose, and allo‐SCT (the latter considered as a time‐dependent variable). We compared data reporting hazard ratio (HR) and corresponding 95% confidence interval (95% CI). To increase the precision of the estimates, the so‐called two‐stage meta‐analysis was performed.^35^ Briefly, the Cox regression model was separately fitted within each region, and between‐region summarized HR were estimated by means of a fixed or random‐effect model, as appropriate.^35^ Incidence rates (IRs) of AP/BP, SPM, and AEs were calculated and expressed ×100 person‐years (p‐y). All analyses were performed using the SAS Statistical Software, version 9.4.

## RESULTS

### Patients' characteristics

Between October 2014 and December 2017, a total of 652 int‐2 and HR MF patients started RUX and entered the study. Region of residence was Lombardy for 290 (44.5%), Lazio for 215 (33%), and Tuscany for 147 (22.5%) of the cases.

The main overall characteristics at the commencement of the RUX therapy divided into region of residence are detailed in Table 1 (with Table S1 reporting these features differentiated by year of start of RUX therapy in Lombardy).

**Table 1:** Characteristics at the start of ruxolitinib therapy of the 652 intermediate‐2 and high‐risk myelofibrosis patients, overall and distinguished by region (Lombardy, Lazio, and Tuscany).

	Total (*N* = 652)	Lombardy (*N* = 290)	Lazio (*N* = 215)	Tuscany (*N* = 147)
*Age, mean (SD), years*	68.8 (10.1)	68.9 (10.0)	68 (10.2)	69.7 (10.3)
*Age class, N (%)*				
<70 years	317 (48.6)	136 (46.9)	119 (55.4)	62 (42.2)
70–79 years	266 (40.8)	125 (43.1)	76 (35.3)	65 (44.2)
≥80 years	69 (10.6)	29 (10.0)	20 (9.3)	20 (13.6)
*Sex, N (%)*				
Males	364 (55.8)	164 (56.6)	114 (53.0)	86 (58.5)
Females	288 (44.2)	126 (43.4)	101 (47.0)	61 (41.5)
*Year of first RUX prescription, N (%)*				
2014	36 (5.5)	32 (11.1)	—	4 (2.7)
2015	238 (36.5)	92 (31.7)	84 (39.1)	62 (42.2)
2016	189 (29)	81 (27.9)	63 (29.3)	45 (30.6)
2017	189 (29)	85 (29.3)	68 (31.6)	36 (24.5)
*MCS class, N (%)*				
Good	159 (24.4)	65 (22.4)	57 (26.5)	37 (25.2)
Intermediate	381 (58.4)	177 (61.0)	121 (56.3)	83 (56.5)
Poor	112 (17.2)	48 (16.6)	37 (17.2)	27 (18.3)
*RUX starting dose, N (%)*				
≥20 mg BID	206 (31.6)	91 (31.4)	80 (37.2)	35 (23.8)
10–15 mg BID	232 (35.6)	108 (37.2)	86 (40.0)	38 (25.9)
5 mg BID	214 (32.8)	91 (31.4)	49 (22.8)	74 (50.3)

Abbreviations: BID, every 12 h; MCS, *Multisource Comorbidity Score*; RUX, ruxolitinib; SD, standard deviation.

In the whole dataset, the mean age at the time of first prescription of RUX was 68.8 (standard deviation, SD: 10.1) years. In detail, 317 (48.6%) subjects were younger than 70 years of age, and 69 (10.6%) were at least 80 years old. Males represented 55.8% (*n* = 364) of the total cohort. The first year of RUX administration was 2014 (5.5%), 2015 (36.5%), 2016 (29%), and 2017 (29%). The MCS class was good in 159 (24.4%), intermediate in 381 (58.4%), and poor in 112 (17.2%) of the subjects. RUX initial dose was at least 20 mg every 12 h (BID) in 206 (31.6%), 10–15 mg BID in 232 (35.6%), and 5 mg BID in 214 (32.8%) subjects (**T1**). Allo‐SCT was performed in 71 (10.9%) patients: 36 (50.7%, detailed in Table S2) in Lombardy, 28 (39.4%) in Lazio, and 7 (9.9%) in Tuscany.

### Overall survival and risk factors for mortality

**Figure 1:**
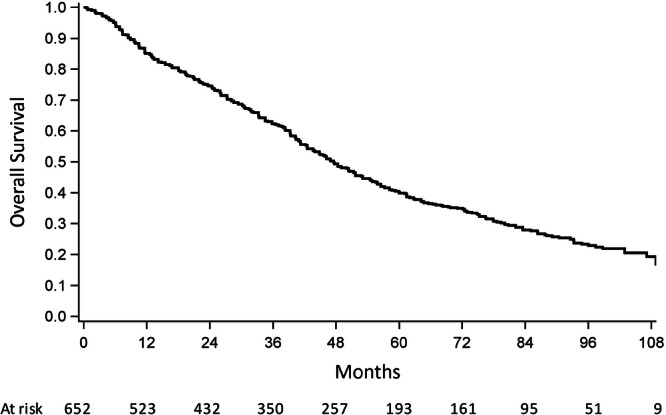
Overall survival of 652 intermediate‐2 and high‐risk myelofibrosis patients treated with ruxolitinib in three Italian regions.

At a median study follow‐up of 36.8 months (range, 0.38–111), 424 (65%) deaths occurred in the overall dataset. Median OS was 48 months (95% CI: 43.2–51.6), as reported in Figure 1. The 1‐, 5‐, and 9‐year survival rates were 85%, 40.3%, and 19.3%, respectively (**F1**). Of note, about 8% of patients had a follow‐up below 6 months because they deceased within that period.

In a multivariable Cox regression model, factors independently associated with increased mortality in the overall cohort were older age classes (70–79 vs. <70 years: HR 2, 95% CI: 1.60–2.49, P < 0.001; ≥80 vs. <70 years: HR 3.31, 95% CI: 2.11–5.19, P < 0.001), poor versus good MCS score (HR 1.97, 95% CI: 1.45–2.68, P < 0.001), and less than 20 mg BID as the initial dose of RUX (HR 1.42, 95% CI: 1.10–1.83, P = 0.007) (Table 2). Female sex was associated with a reduced risk of death in the same analysis (HR 0.63, 95% CI: 0.45–0.89; P = 0.008) (**T2**). As for the Lombardy dataset, additional information on mortality rates based on a more detailed categorization of patients by age classes is shown in Table S3.

**Table 2:** A multivariable Cox regression model evaluating risk factors for mortality in 652 intermediate‐2 and high‐risk myelofibrosis patients treated with ruxolitinib.

*Variable*	*Category*	*HR (95% CI)*	P
Sex	Females (vs. males)	0.63 (0.45–0.89)	**0.008**
Age class	70–79 (vs. <70) years	2.00 (1.60–2.49)	**<0.001**
	≥80 (vs. <70) years	3.31 (2.11–5.19)	**<0.001**
Year of first RUX dose	2014 (vs. 2015)	1.24 (0.81–1.89)	0.329
	2016 (vs. 2015)	0.84 (0.63–1.12)	0.238
	2017 (vs. 2015)	0.96 (0.62–1.47)	0.843
MCS class	Intermediate (vs. good)	1.27 (0.99–1.64)	0.059
	Poor (vs. good)	1.97 (1.45–2.68)	**<0.001**
Starting RUX dose	<20 (vs. ≥20) mg BID	1.42 (1.10–1.83)	**0.007**
Allo‐SCT	Yes (vs. no)	1.09 (0.44–2.69)	0.856

*Note:* Bold values indicate statistically significant.

Abbreviations: allo‐SCT, allogeneic hematopoietic stem cell transplantation; BID, every 12 h; CI, confidence interval; HR, hazard ratio; MCS, *Multisource Comorbidity Score*; RUX, ruxolitinib.

Looking at the 71 subjects who received allo‐SCT, 41 (57.7%) deceased, with a median OS after allo‐SCT of 44.4 months (95% CI: 24.0–not reached; Figure S1).

### Time to treatment discontinuation

**Figure 2:**
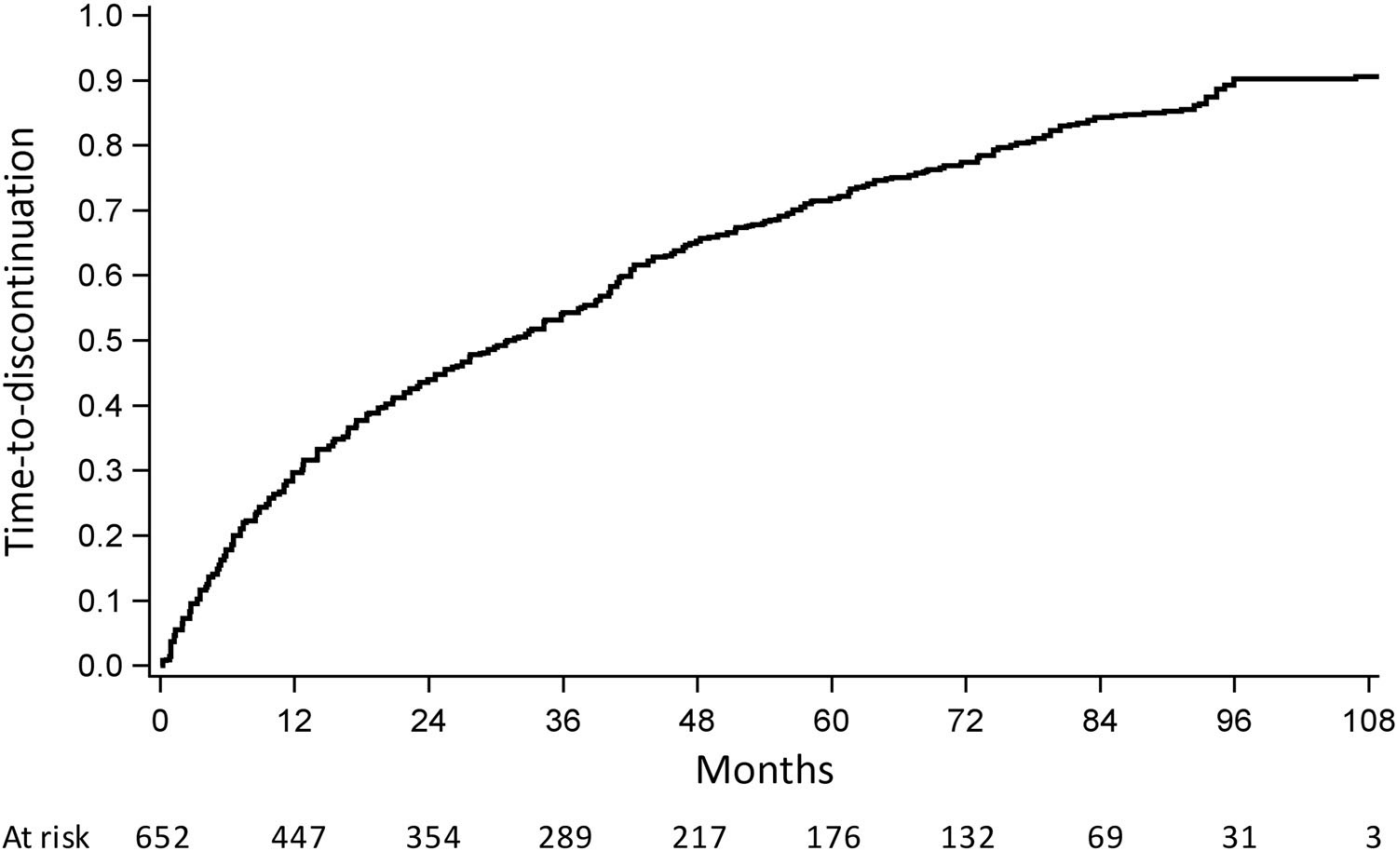
Time to treatment discontinuation in 652 intermediate‐2 and high‐risk myelofibrosis patients treated with ruxolitinib in three Italian regions.

Out of 652 higher‐risk MF patients, TD was reported in 537 (82.4%): 253 (87.2%) in Lombardy, 165 (76.7%) in Lazio, and 119 (80.9%) in Tuscany. Median TTD was 31.2 months (95% CI: 26.4–36.0; Figure 2). The 1‐, 5‐, and 9‐year TD rates were 29.7%, 71.8%, and 90.6%, respectively (**F2**). Figure S2 describes the survival estimate of 140 (55.3%) patients who discontinued RUX for reasons different from death in Lombardy (median OS 9.6 months, 95% CI: 7.4–13.4).

### Changes in RBC units' burden during the first 6 months of RUX treatment

Out of 594 (91.1%) patients with at least 6 months of follow‐up, the number of RBC units received at baseline was zero in 490 (82%, RBC0), one to five in 82 (14%, RBC1–5), and at least six in 22 (4%, RBC6) (Table 3).

**Table 3:** Changes in red blood cell units number requirement in the first 6 months of ruxolitinib treatment compared to baseline in 594 intermediate‐2 and high‐risk myelofibrosis patients.

		Period of 6 months following the start of RUX therapy			
		*No RBC U, N (%)*	*1–5 RBC U, N (%)*	*≥6 RBC U, N (%)*	Total, *N* (%)
**Period of 3 months before RUX start**	***No RBC U, N (%)***	376 (63.3)	113 (19.0)	1 (0.2)	490 (82)
	***1–5 RBC U, N (%)***	29 (4.9)	48 (8.1)	5 (0.8)	82 (14)
	***≥6 RBC U, N (%)***	3 (0.5)	11 (1.9)	8 (1.3)	22 (4)
**Total,** ***N*** **(%)**		408 (69)	172 (29)	14 (2)	594

Abbreviations: RBC U, red blood cell unit; RUX, ruxolitinib.

During the first 6 months of RUX treatment, 376 (76.7%) of RBC0 subjects continued to be free from RBC transfusions, while 113 (23.1%) received one to five units, and one (0.2%) at least six RBC units (**T3**). In the same timeframe, 29 (35.4%) RBC1–5 and 3 (13.6%) RBC6 cases did not receive any RBC unit (**T3**). Lastly, 48 (58.5%) RBC1–5 and 8 (36.4%) of RBC6 patients received the same number of RBC units in the first 6 months of RUX compared to baseline (**T3**).

### Incidence of adverse events, secondary malignancies, and clonal progression

Table 4 summarizes the frequency of AEs, SPM, and clonal progression (meant as evolution into AP/BP) in the overall dataset, which required hospital admission during study follow‐up.

**Table 4:** Frequency and incidence of adverse events, secondary malignancies, and clonal evolution in 652 intermediate‐2 and high‐risk myelofibrosis patients treated with ruxolitinib.

Event	*N* (%)	Incidence rate (×100 p‐y)
Infections	182 (27.9)	10.30
Bleeding	65 (10)	3.47
Thrombosis	29 (4.4)	1.56
Splenectomy	14 (2.1)	0.68
Solid tumors	118 (18.1)	5.47
Hematological complications except LPD	65 (10)	2.82
LPD	25 (3.8)	1.07
AP/BP	110 (16.9)	5.22

Abbreviations: AP, accelerated phase; BP, blast phase; LPD, lymphoproliferative disorder; p‐y, patient‐years.

The most frequent AEs were infections, which occurred in 182 (27.9%) cases with an IR of 10.3 × 100 p‐y, followed by bleeding events (*n* = 65, 10%, IR: 3.47 × 100 p‐y) and thrombosis (*n* = 29, 4.4%, IR: 1.56 × 100 p‐y) (**T4**).

Of note, 118 (18.1%) developed a solid tumor, 65 (10%) a hematological complication excluded a lymphoproliferative disorder (LPD), and 25 (3.8%) an incidental LPD, with a corresponding IR of 5.47, 2.82, and 1.07 × 100 p‐y (**T4**). The time between the first prescription of RUX and a diagnosis of a solid tumor in the Lombardy subgroup is described in Table S4.

Evolution into AP/BP was reported in 110 (16.9%) patients of the total cohort, with an IR of 5.22 × 100 p‐y (**T4**). Table S5 reports information on the above events distinguished by region, while Table S6 describes the type and incidence of infections, bleeding events, thrombosis, and solid tumors in our study population.

### Costs analysis

In the overall cohort, the individual average cost rate by year was €30,675: €25,860 (84.3%) related to RUX supply, €2941 (9.6%) for hospitalizations, €1772 (5.8%) for outpatient visits, and €102 (0.3%) for emergency department (ED) accesses (Table 5).

**Table 5:** Average annual cost rate per person in 652 intermediate‐2 and high‐risk myelofibrosis patients treated with ruxolitinib, in the overall dataset, based on age and on the Multisource Comorbidity Score classes.

	Average annual cost rate per person (€)						
	Overall cohort (*n* = 652)	<70 years (*n* = 317)	70–79 years (*n* = 266)	≥80 years (*n* = 69)	Good MCS score (*n* = 159)	Intermediate MCS score (*n* = 381)	Poor MCS score (*n* = 112)
Inpatient admission	2941	2543	3364	3666	2082	2916	5064
Emergency department access	102	71	144	141	74	105	161
RUX supply	25,860	26,553	25,543	22,520	25,014	26,200	26,171
Outpatient evaluation	1772	1831	1756	1432	1776	1751	1865
Total	30,675	30,998	30,807	27,759	28,946	30,972	33,261

Abbreviations: MCS, *Multisource Comorbidity Score*; RUX, ruxolitinib.

Considering the three age classes, the yearly average cost rate was €30,998 for patients younger than 70 years, €30,807 for those aged 70–79 years, and €27,759 for patients at least 80 years old (**T5**). Of note, the costs for RUX supply were higher for progressively younger cases, while expenses for inpatient admissions and ED accesses were more relevant for older ones (**T5**).

Lastly, the annual cost of RUX resulted in €28,946, €30,972, and €33,261 for subjects with good, intermediate, and poor MCS scores, respectively (**T5**). In addition, Table S7 replicates the above analysis considering the three regional data subsets.

## DISCUSSION

The treatment landscape of MF has dramatically changed in the last decade, with the introduction in the clinical practice of JAKis, in particular of RUX.^2^ RWD on the prolongation of survival with RUX^12^, ^13^, ^14^, ^15^, ^16^, ^17^ and on the benefit for patients who achieved a clinical improvement before SCT are available.^5^

Using RWD could have an enormous impact to help investigators and health policy makers develop better ways to treat affected populations, ultimately enhancing the quality of care.^33^, ^34^ Limitations of RWD capturing are well‐known,^36^ however, they can offer a continuous learning of diseases and drugs. In this study, we evaluated HCUD obtained from healthcare systems of three Italian regions to inform outcomes of patients with higher‐risk MF, receiving RUX treatment. We analyzed an unselected large population‐based cohort of 652 int‐2 and HR MF subjects with available follow‐up.

At study entry, the mean age of our cohort was around 68 years, which roughly corresponds to the average age of patients affected by MF who receive RUX in clinical trials.^2^, ^37^, ^38^ Around 40% and 10% of included cases were at least 70 and 80 years old, respectively, suggesting that age per se is not considered a limit for starting RUX outside clinical studies.^39^ Similarly, the clinical profile, measured through the MCS score, was intermediate in 58.4% and poor in 17.2% of the subjects, underlying that RUX can also be used in patients with numerous comorbidities due to its well‐characterized and acceptable safety profile.^39^, ^40^

In our dataset, the initial dose of RUX was found to be almost equally distributed among 5, 10–15 mg BID, and at least 20 mg BID. As per label indications, the starting dose of RUX should be chosen according to platelet (PLT) count. In the registrational trials regarding int‐2 and HR patients with MF, only patients with PLT ≥ 100 × 10^9^/L could be included, and therefore, high doses of RUX were studied.^2^ Looking at our dose analysis, the Tuscany Region seems to have a high rate of patients receiving lower doses of RUX. This could be potentially related to a significant proportion of cytopenic patients.^41^ However, different RW studies have shown that physicians frequently start RUX at a reduced dose compared to that per label, despite accumulating evidence on a relationship between appropriate starting dosage and better OS.^15^, ^16^, ^17^

We then observed in the Lombardy subcohort that the rate of subjects receiving higher starting doses of RUX numerically decreased over time, while that of patients treated with lower doses increased. As we presumed that we managed patients with a more aggressive disease, left untreated till RUX approval, at the beginning, one can assume that the increasing rate of lower starting doses of RUX was just related to the expected hematological toxicity of the drug.

In this population of 652 int‐2 and HR MF patients, observed for a median follow‐up period of 3 years, the median OS was 4 years. This figure appears worse with respect to that of the post hoc analysis of the COMFORT trials,^10^, ^11^ reporting a median OS of 5.3 years in around 300 higher‐risk MF.^10^, ^11^ Comorbidity profiles and risk category composition could explain this difference.^10^, ^11^ In the Phase IIIb expanded access JUMP study with more relaxed entry criteria, the median OS was 4.9 and 2.8 years among 755 int‐2 and 194 HR MF patients, respectively—results closer to our data. Out of 1010 MF subjects of the European ERNEST registry, 108 received RUX, subsequently entering a propensity score matching analysis with hydroxyurea.^12^ After a median follow‐up of around 5 years, median OS was 6.7 years in RUX‐treated patients; however, this cohort included subjects from low to high‐risk DIPSS.^12^ A single‐center experience of 844 MF patients reported that the outcome of those at higher DIPSS risk (around half of the cohort) improved significantly after 2011, which corresponded to the year of RUX approval in the United States (median OS 3.8 vs. 2.4 years in 2000–2010).^13^ Actually, the outcome was mostly favorable for RUX‐exposed cases.^13^ In a retrospective analysis of the US Medicare Fee‐for‐service claims database looking at contemporary intermediate‐1 to HR MF cases, the median OS of 272 RUX‐exposed patients was not reached, while it was 3.7 years for 1127 subjects who never received the drug despite its approval.^14^

In our dataset, factors independently associated with mortality were older age classes, poor MCS score, and less than 20 mg BID as RUX initial dose, while a protective role was covered by female sex. In a post hoc analysis of the COMFORT trials,^10^ investigators underlined the detrimental role of older baseline age and the favorable impact on survival of female sex. In other MPN cohorts, women showed a better prognosis^42^: the reasons are not completely understood, but sex hormones and telomeres length could be implied.^42^ In a European analysis of around 400 RUX‐treated MF patients,^40^ 41% had a high Charlson Comorbidity Index (CCI), with a DIPSS‐adjusted OS significantly reduced for those with increasing CCI.^40^ The relevance of RUX dose on outcome has been clearly demonstrated in the ambispective observational RUXO‐REL study,^17^ with 20 mg BID given during the first 6 months of treatment as a cutoff.

Recently, Breccia et al.^15^ have published a large Italian cohort of 3494 MF patients identified by the *Agenzia Italiana del Farmaco* (AIFA) monitoring registries. In that series, 7.5% and 63% of cases were at int‐1 and int‐2 risk, respectively.^15^ At a minimum follow‐up of 3 years, the median OS was 6.5 years for patients who started at a full RUX dose (33%), while it was significantly worse (4.4 years) in the case of a reduced dosage (67%).^15^ This was true also when restricting the analysis to int‐2 and HR subsets.^15^ In the interim analysis of the observational ROMEI study, 306 MF cases (70% at higher IPSS risk) were included.^16^ The rate of patients that started RUX at per label dose was higher, being 57%.^16^ The latter group showed a significant survival improvement compared to those receiving a “lower than expected” starting dose (median OS, not reached vs. 4.7 years).^16^ Besides, both clinical trials^43^ and the RWD^17^, ^44^, ^45^ have highlighted the correlation between higher RUX doses (at treatment start and/or in the first months) and spleen response, with the latter being associated with better outcome.

Considering the mean age of our cohort and the limited number of cases with a good comorbidity profile (24%), only 10% of our higher‐risk patients underwent allo‐SCT after commencing RUX. Besides, around 58% deceased after the procedure with a median OS of 3.7 years. In the dataset used for generating the *Myelofibrosis Transplant Scoring System* (MTSS),^6^ the 5‐year OS after allo‐SCT of 205 MF patients (over half at higher risk) was around 60%. Information on the MTSS score was not available in our study, but we cannot exclude that patients' selection criteria were not consistent.

Treatment discontinuation was reported in around 80% of our cohort overt time, with a median TTD of 2.6 years, similar to the registrational studies (50% and 75% by 3 and 5 years, respectively).^2^, ^11^ Out of 524 MF patients treated in 20 European centers, 41% stopped RUX at 3 years, with having an int‐2/HR DIPSS score among baseline predictors of drug discontinuation.^27^

In our cohort, 82% of patients did not need RBC transfusions before starting RUX (RBC0). We found that—during the first 6 months of treatment—77% of baseline RBC0, 35% of RBC1–5, and 14% of RBC6 were RBC‐units free. Overall, 69% did not receive any RBC unit in this timeframe, like the registrational trials of higher‐risk cases.^2^ In a post hoc analysis of the JUMP study,^46^ around half of both non‐anemic and anemic patients at baseline did not develop new‐onset or worsening anemia up to Week 12. In a recent report of the RUXO‐REL study,^20^ a major anemia response^47^ at 6 months was achieved in 4 (5%) of 80 baseline anemic cases, but interestingly 3 out of 4 of them were RBC‐transfusion‐dependent at the start of RUX therapy.^20^ In this setting, RWD provide useful information for the management of MF. It is well known that RUX is not an anemia‐sparing drug,^2^ but other JAKis (such as MMB or pacritinib) and innovative molecules under development have higher potential to improve anemia.^48^

Concerning AEs collected via HCUD, we had availability only of those that led to hospital admission. This limitation can especially underdetect the number of mild‐moderate infections that can be managed at home. However, infections account for an IR of 10.3 × 100 p‐y in our study. This is not dissimilar from data which came from a European study of 446 MF cases (>80% int‐2 and HR) exposed to RUX for a median time of 2 years, reporting an IR of infectious events of 17 × 100 p‐y.^22^ Additionally, different ages, rates of higher‐risk patients, and comorbidities are important variables across studies that can explain the different numbers reported. We investigated SPM as one of the most critical events during the treatment of patients with chronic diseases such as MF for patients, doctors, and health policy makers. In our collection, we can identify all types of tumors occurring during follow‐up in each patient treated with RUX. We found that solid tumors had a higher incidence (5.47 × 100 p‐y) than other hematological complications and LPD (2.82 and 1.07 × 100 p‐y, respectively). We confirmed the incidence of SPM obtained in other series,^21^, ^23^, ^24^ highlighting the robustness of this RWD collection, avoiding the cost of clinical trials. Of note, NMSC appear to be the most frequent type of solid tumors being diagnosed in RUX‐treated MF patients (7.7%), in line with existing literature.^23^, ^24^, ^25^ MF progression rate into AP/BP involved 16.9% of patients with an IR of 5.22 × 100 p‐y. Among 886 RUX‐treated MF patients (half at higher risk), included in the RUX‐MF retrospective study, BP incidence was 3.74 × 100 p‐y.^4^ Our current results are aligned with the aforementioned analysis, and they confirm that RUX does not impact the probability of BP evolution in MF.

Finally, we obtained data on the costs of RUX in Italy, which is useful both for health policy makers and for comparison among different health systems. The average annual cost rate of RUX per person in the analyzed regions has been estimated at €30,675. Using healthcare claims data from the IQVIA PharMetrics Plus database, Liu et al.^30^ have recently reported that patients with anemia before RUX treatment exhibited higher median annual all‐cause healthcare resource utilization, in terms of inpatient and ED admissions, outpatient visits, and all‐cause total healthcare costs compared with nonanemic patients.^30^ Of note, anemic subjects were older and had a higher mean CCI.^30^ Considering anemic cases, a cost‐comparison model was recently developed in the United States for patients treated with either RUX or MMB as first line.^31^ Substantial savings were evident with RUX due to its lower pharmacy costs, despite estimated higher transfusion expenses.^31^ Anyway, the latter could not be entirely captured in this type of analysis.^31^ Merging data from clinical trials on MMB and the US IBM MarketScan Commercial database, Masarova et al.^49^ demonstrated cost savings for MMB versus RUX in anemic patients, including the RBC‐transfusion‐dependent ones.

Despite the limitation of missing details on disease characteristics (i.e., blood counts, mutation profile, karyotype, and bone marrow fibrosis), this novel HCUD‐based approach on large, updatable cohorts of MF patients with available follow‐up informs the outcome of RUX treatment. Data of this type are crucial for improving our understanding of drug usage in real‐world settings as well as for pharmacoeconomic considerations.

## AUTHOR CONTRIBUTIONS

**Barbara Mora**: Conceptualization; writing—original draft; writing—review and editing. **Matteo Franchi**: Methodology; formal analysis; writing—review and editing. **Ludovica Margotto**: Methodology; formal analysis; writing—review and editing. **Olivia Leoni**: Data curation; writing—review and editing. **Daniela D'ippoliti**: Writing—review and editing; data curation. **Emanuela Carloni**: Data curation; writing—review and editing. **Ilaria Cozzi**: Writing—review and editing; data curation. **Enrica Santelli**: Writing—review and editing; data curation. **Fabrizio Gemmi**: Writing—review and editing; data curation. **Claudia Szasz**: Data curation; writing—review and editing. **Margherita Maffioli**: Writing—review and editing. **Carmelo Gurnari**: writing—review and editing. **Enrico Attardi**: Writing—review and editing. **Daniele Cattaneo**: Writing—review and editing. **Marta Bortolotti**: Writing—review and editing. **Nicola Stefano Fracchiolla**: Writing—review and editing. **Alessandra Iurlo**: Writing—review and editing. **Giovanni Corrao**: Writing—review and editing. **Matteo Giovanni Della Porta**: Writing—review and editing. **Alessandro Maria Vannucchi**: Writing—review and editing. **Maria Teresa Voso**: Conceptualization; writing—review and editing. **Paola Guglielmelli**: Writing—review and editing; conceptualization. **Francesco Passamonti**: Conceptualization; writing—original draft; writing—review and editing.

## CONFLICT OF INTEREST STATEMENT

Barbara Mora received fees for lectures from Incyte, Novartis, and GSK, and for advisory board from GSK.

Alessandro Maria Vannucchi received fees for lectures and advisory boards from Novartis, Incyte, GSK, AbbVie, AOP, Blueprint, Menarini Stemline, and Italfarmaco.

Paola Guglielmelli received fees for lectures and advisory boards from Novartis and GSK.

Francesco Passamonti received honoraria for lectures from Novartis, Bristol‐Myers Squibb, GSK, AbbVie, Jazz, Janssen, AOP, and Menarini Stemline, and for advisory boards from Novartis, Bristol‐Myers Squibb, GSK, AbbVie, Keros, Takeda, and Sumitomo.

For Matteo Franchi, Ludovica Margotto, Olivia Leoni, Daniela D'ippoliti, Emanuela Carloni, Ilaria Cozzi, Enrica Santelli, Fabrizio Gemmi, Claudia Szasz, Margherita Maffioli, Carmelo Gurnari, Enrico Attardi, Daniele Cattaneo, Marta Bortolotti, Nicola Stefano Fracchiolla, Alessandra Iurlo, Giovanni Corrao, Matteo Giovanni Della Porta, and Maria Teresa Voso, no relevant conflicts of interest were declared.

## ETHICS STATEMENT

The Institutional Review Board of Insubria (Varese, Italy) approved the study, which was conducted in accordance with the Declaration of Helsinki.

## FUNDING

B.M., F.P., M.G.D.P., A.M.V., P.G., and M.T.V. were funded by Ministero della Salute, Rome, Italy (Finalizzata 2018, NET‐2018‐12365935, Personalized medicine program on myeloid neoplasms: characterization of the patient's genome for clinical decision making and systematic collection of real‐world data to improve quality of health care). F.P. and M.B. have been supported by grants from Fondazione Matarelli, Milan, Italy. M.F. and L.M. were funded by the Italian Ministry of University and Research (PRIN 2022 ‐ Real‐world evaluation of cancer outcomes by integrating administrative and hospital‐based health‐related data: the We‐Care project, grant number 2022YCMF4). Open access publishing facilitated by Universita degli Studi di Milano, as part of the Wiley ‐ CRUI‐CARE agreement.

## Supporting information

Supporting Information.

## Data Availability

The dataset analyzed during the current study is available by mailing the corresponding author on reasonable request.
