# Supplementary material for: Health Care Utilization Databases obtained from health system inform outcome for ruxolitinib treatment in patients with myelofibrosis
Source: Hemasphere. 2026 Feb 24;10(2):e70316. doi: 10.1002/hem3.70316 (PMC12931254; doi:10.1002/hem3.70316)

**Supplementary Information**

**Health Care Utilization Databases obtained from Health System inform outcome for ruxolitinib treatment in patients with myelofibrosis.**

**Supplementary Methods.**

**Study populations and covariates**

The *Multisource Comorbidity Score* (MCS) is a synthetic score of comorbidities developed and validated in Italy.^1,2^ In this study, the MCS is based on inpatients diagnostic codes and drug prescriptions collected within two years before starting RUX. Patients were classified as having a good (score, 0 to 4), intermediate (5 to 14) and poor (at least 15) clinical profile according to MCS.^1,2^

**Statistical analyses**

To increase the precision of the overall and the time to treatment failure survival estimates, between-region summarized Kaplan-Meier (KM) curves were used.^3^ As regional data were not available to be analyzed in a pooled analysis, a method for reconstructing individual-patient data starting from each regional KM curve was applied.^3^ Briefly, a digital software was used to read the coordinates of the KM curves within each region.^3^ Information on the number of patients still at risk at each year of follow-up and the total number of outcome events was used to solve the inverted KM equation, which allowed for reconstructing the regional KM data for each arm, thus obtaining pooled individual-patient data.^3^

**REFERENCES**

1) Corrao G, Rea F, Di Martino M, et al. Developing and validating a novel multisource comorbidity score from administrative data: a large population-based cohort study from Italy. *BMJ Open*. 2017;7(12):e019503.

2) Corrao G, Rea F, Carle F, et al. Measuring multimorbidity inequality across Italy through the multisource comorbidity score: a nationwide study. *Eur J Public Health*. 2020;30(5):916-921.

3) Guyot P, Ades AE, Ouwens MJ, Welton NJ. Enhanced secondary analysis of survival data: reconstructing the data from published Kaplan-Meier survival curves. *BMC Med Res Methodol*. 2012;12:9.

**Table S1. Characteristics of the 290 intermediate-2 and high risk myelofibrosis patients of the Lombardy region, stratified by the year of start of ruxolitinib therapy.**

|  |  |  |  |  | **P** |
| --- | --- | --- | --- | --- | --- |
|  | **2014**  **(n=32)** | **2015**  **(n=92)** | **2016**  **(n=81)** | **2017**  **(n=85)** |  |
| **Age, mean (SD)**, years | 66.1 (8.3) | 69.9 (9.0) | 67.5 (10.2) | 70.0 (11.1) | 0.112 |
| **Age class, N (%)** |  |  |  |  |  |
| <70 years | 20 (62.5) | 37 (40.2) | 43 (53.1) | 36 (42.3) | 0.088 |
| 70-79 years | 12 (37.5) | 46 (50.0) | 31 (38.3) | 36 (42.3) |  |
| ≥80 years | 0 (0.0) | 9 (9.8) | 7 (8.6) | 13 (15.4) |  |
| **Sex, N (%)** |  |  |  |  |  |
| Males | 21 (65.6) | 51 (55.4) | 44 (54.3) | 48 (56.5) | 0.733 |
| Females | 11 (34.4) | 41 (44.6) | 37 (45.7) | 37 (43.5) |  |
| **MCS class, N (%)** |  |  |  |  |  |
| Good | 11 (34.4) | 15 (16.3) | 23 (28.4) | 16 (18.8) | 0.246 |
| Intermediate | 17 (53.1) | 61 (66.3) | 43 (53.1) | 56 (65.9) |  |
| Poor | 4 (12.5) | 16 (17.4) | 15 (18.5) | 13 (15.3) |  |
| **RUX starting dose, N (%)** |  |  |  |  |  |
| ≥ 20 mg BID | 14 (43.8) | 29 (31.5) | 20 (24.7) | 28 (32.9) | 0.349 |
| 10-15 mg BID | 10 (31.2) | 31 (33.7) | 38 (46.9) | 29 (34.2) |  |
| 5 mg BID | 8 (25.0) | 32 (34.8) | 23 (28.4) | 28 (32.9) |  |

**Legend:** SD= standard deviation; RUX= ruxolitinib; MCS= *Multisource Comorbidity Score*; BID= every 12 hours.

**Table S2. Characteristics at the time of start of ruxolitinib of the 36 intermediate-2 and high-risk myelofibrosis patients of the Lombardy region that received an allogeneic stem cell transplantation.**

|  | **Patients**  **(n=36)** |
| --- | --- |
| **Age, mean (SD),** years | 54.4 (9.4) |
| **Age class, N (%)** |  |
| <60 years | 25 (69.4) |
| 60-64 years | 6 (16.7) |
| 65-69 years | 5 (13.9) |
| **Sex, N (%)** |  |
| Males | 22 (61.1) |
| Females | 14 (38.9) |
| **Year of first RUX prescription, N (%)** |  |
| 2014 | 2 (5.6) |
| 2015 | 9 (25.0) |
| 2016 | 14 (38.8) |
| 2017 | 11 (30.6) |
| **MCS class, N (%)** |  |
| Good | 13 (36.1) |
| Intermediate | 21 (58.3) |
| Poor | 2 (5.6) |
| **RUX starting dose, N (%)** |  |
| ≥ 20 mg BID | 11 (30.6) |
| 10-15 mg BID | 11 (30.6) |
| 5 mg BID | 14 (38.8) |

**Legend:** SD= standard deviation; RUX= ruxolitinib; MCS= *Multisource Comorbidity Score*; BID= every 12 hours.

**Table S3. Mortality rate of the 290 intermediate-2 and high-risk myelofibrosis patients of the Lombardy region treated with ruxolitinib, based on different age classes.**

| **Age class, years** | **Patients, n** | **Deaths, n** | **Person-years*** | **Mortality rate (x 1000 person-years) with 95% CI** |
| --- | --- | --- | --- | --- |
| **<60** | 45 | 8 | 126.3 | 63.3 (29.4-120.3) |
| **60-64** | 31 | 16 | 144.4 | 110.8 (65.6-176.1) |
| **65-69** | 60 | 45 | 258.9 | 173.8 (128.3-230.5) |
| **70-74** | 66 | 56 | 268.0 | 209.0 (159.4-269.4) |
| **75-79** | 59 | 54 | 161.9 | 333.5 (253.1-431.9) |
| **≥80** | 29 | 28 | 76.5 | 366.0 (248.0-521.9) |

**Legend:** *censored at allogeneic stem cell transplantation; 95% CI: 95% Confidence Intervals.

**Table S4. Time between the first prescription of ruxolitinib and diagnosis of a solid tumor in 49 intermediate-2 and high-risk myelofibrosis patients of the Lombardy region.**

| **Time, months** | **Patients, n (%)** |
| --- | --- |
| **<6** | 8 (16.3) |
| **6-12** | 8 (16.3) |
| **13-36** | 12 (24.5) |
| **37-60** | 14 (28.6) |
| **>60** | 7 (14.3) |

**Table S5.** **Frequency and incidence of adverse events, secondary malignancies, and clonal evolution in 652 intermediate-2 and high-risk myelofibrosis patients treated with ruxolitinib distinguished by Region (Lombardy, Tuscany, Lazio).**

|  | **Lombardy** | | **Tuscany** | | **Lazio** | |
| --- | --- | --- | --- | --- | --- | --- |
| **Event** | **N (%)** | **Incidence rate**  **(x100 p-y)** | **N (%)** | **Incidence rate**  **(x100 p-y)** | **N (%)** | **Incidence rate**  **(x100 p-y)** |
| **Infections** | 106 (36.6) | 14.57 | 42 (28.6) | 12.83 | 34 (15.8) | 4.77 |
| **Bleeding** | 34 (11.7) | 4.28 | 14 (9.5) | 3.98 | 17 (7.9) | 2.34 |
| **Thrombosis** | 17 (5.9) | 2.24 | 9 (6.1) | 2.51 | 3 (1.2) | 0.41 |
| **Splenectomy** | 8 (2.8) | 0.96 | 4 (2.7) | 1.10 | 2 (0.9) | 0.23 |
| **Solid tumours** | 49 (16.9) | 5.27 | 33 (22.4) | 7.65 | 36 (16.7) | 4.53 |
| **Haematological complications except LPD** | 37 (12.8) | 3.79 | 19 (12.9) | 4.12 | 9 (4.2) | 1.04 |
| **LPD** | 15 (5.2) | 1.50 | 2 (1.4) | 0.41 | 8 (3.7) | 0.94 |
| **AP/BP** | 62 (21.4) | 7.32 | 24 (16.3) | 5.13 | 24 (11.2) | 3.03 |

**Legend:** LPD= Lymphoproliferative disorders; AP= accelerated phase; BP= blast phase; p-y= patient-years.

**Table S6.** **List of infections, haemorrhagic events, thrombosis and solid tumor types in 652 intermediate-2 and high-risk myelofibrosis patients treated with ruxolitinib.**

| **Event** | **N (%)** |
| --- | --- |
| **Infections**  Pneumonia  Blood stream  Bacterial, nos  Nos  COVID-19  Genitourinary system  Viral, nos  Viral hepatitis  Mycobacterial  Fungal, nos | 182 (27.9)  65 (35.8)  39 (21.4)  19 (10.5)  16 (8.8)  15 (8.2)  15 (8.2)  7 (3.8)  2 (1.1)  2 (1.1)  2 (1.1) |
| **Haemorrhagic events**  Gastrointestinal  Nos  Cerebral  Hematuria  Hemoptysis  Retinal | 65 (10)  30 (46.2)  19 (29.2)  11 (16.9)  3 (4.7)  1 (1.5)  1 (1.5) |
| **Thrombosis**  Venous thromboembolism  Ischemic heart disease  Cerebrovascular ischemia  Cerebral venous thrombosis  Peripheral artery disease  Splanchnic venous thrombosis | 29 (4.4)  11 (37.9)  6 (20.7)  6 (20.7)  3 (10.4)  2 (6.9)  1 (3.4) |
| **Solid tumors**  Non melanoma skin cancers  Nos  Respiratory tract  Gastrointestinal tract  Liver  Urogenital tract  Head and neck  Melanoma  Breast  Thyroid | 118 (18.1)  50 (42.4)  40 (33.4)  7 (6)  5 (4.2)  5 (4.2)  4 (3.5)  2 (1.8)  2 (1.8)  2 (1.8)  1 (0.9) |

**Legend:** Nos= not otherwise specified.

**Table S7.** **Average annual cost rate *per* person in 652 intermediate-2 and high risk myelofibrosis patients treated with ruxolitinib, based on Region, age class and on the *Multisource Comorbidity Score* classes.**

|  | **Average annual cost rate *per* person (€) in Lombardy** | | | | | | |
| --- | --- | --- | --- | --- | --- | --- | --- |
|  | **Overall** | **<70 years** | **70-79 years** | **≥80 years** | **Good MCS score** | **Intermediate MCS score** | **Poor MCS score** |
|  | **(n = 290)** | **(n = 136)** | **(n = 125)** | **(n = 29)** | **(n = 65)** | **(n = 177)** | **(n = 48)** |
| **In patient admission** | 3508 | 3409 | 3588 | 3758 | 2559 | 3293 | 6348 |
| **Emergency department access** | 157 | 107 | 209 | 226 | 131 | 151 | 239 |
| **RUX supply** | 29,040 | 30,373 | 28,229 | 24,198 | 28,752 | 29,137 | 29,116 |
| **Outpatient evaluation** | 2591 | 2676 | 2517 | 2409 | 2785 | 2533 | 2504 |
| **Total** | 35,296 | 36,565 | 34,543 | 30,591 | 34,227 | 35,114 | 38,207 |
|  | **Average annual cost rate *per* person (€) in Lazio** | | | | | | |
|  | **Overall** | **<70 years** | **70-79 years** | **≥80 years** | **Good MCS score** | **Intermediate MCS score (n = 121)** | **Poor MCS score** |
|  | **(n = 215)** | **(n = 119)** | **(n = 76)** | **(n = 20)** | **(n = 57)** |  | **(n = 37)** |
| **In patient admission** | 1797 | 1432 | 2333 | 2770 | 1480 | 1989 | 1769 |
| **Emergency department access** | 22 | 15 | 33 | 46 | 19 | 20 | 44 |
| **RUX supply** | 23,402 | 24,253 | 22,338 | 19,964 | 21,433 | 24,623 | 23,125 |
| **Outpatient evaluation** | 1133 | 1220 | 1032 | 742 | 1304 | 1032 | 1136 |
| **Total** | 26,354 | 26,920 | 25,736 | 23,522 | 24,236 | 27,664 | 26,074 |
|  | **Average annual cost rate *per* person (€) in Tuscany** | | | | | | |
|  | **Overall** | **<70 years** | **70-79 years** | **≥80 years** | **Good MCS score** | **Intermediate MCS score** | **Poor MCS score** |
|  | **(n = 147)** | **(n = 62)** | **(n = 65)** | **(n = 20)** | **(n = 37)** | **(n = 83)** | **(n = 27)** |
| **In patient admission** | 3586 | 2895 | 4242 | 4164 | 2337 | 3489 | 7026 |
| **Emergency department access** | 120 | 102 | 149 | 99 | 73 | 134 | 165 |
| **RUX supply** | 23,151 | 22,721 | 23,976 | 22,174 | 24,945 | 22,063 | 24,099 |
| **Outpatient evaluation** | 1083 | 1197 | 1068 | 685 | 856 | 1105 | 1515 |
| **Total** | 27,940 | 26,915 | 29,435 | 27,122 | 28,211 | 26,791 | 32,805 |

**Legend:** RUX= ruxolitinib; MCS= *Multisource Comorbidity Score*.

**Figure S1. Overall survival of 71 intermediate-2 and high-risk myelofibrosis patients treated with ruxolitinib in three Italian Regions that received an allogeneic stem cell transplantation.**

At risk

71

47

34

28

18

15

10

7

0


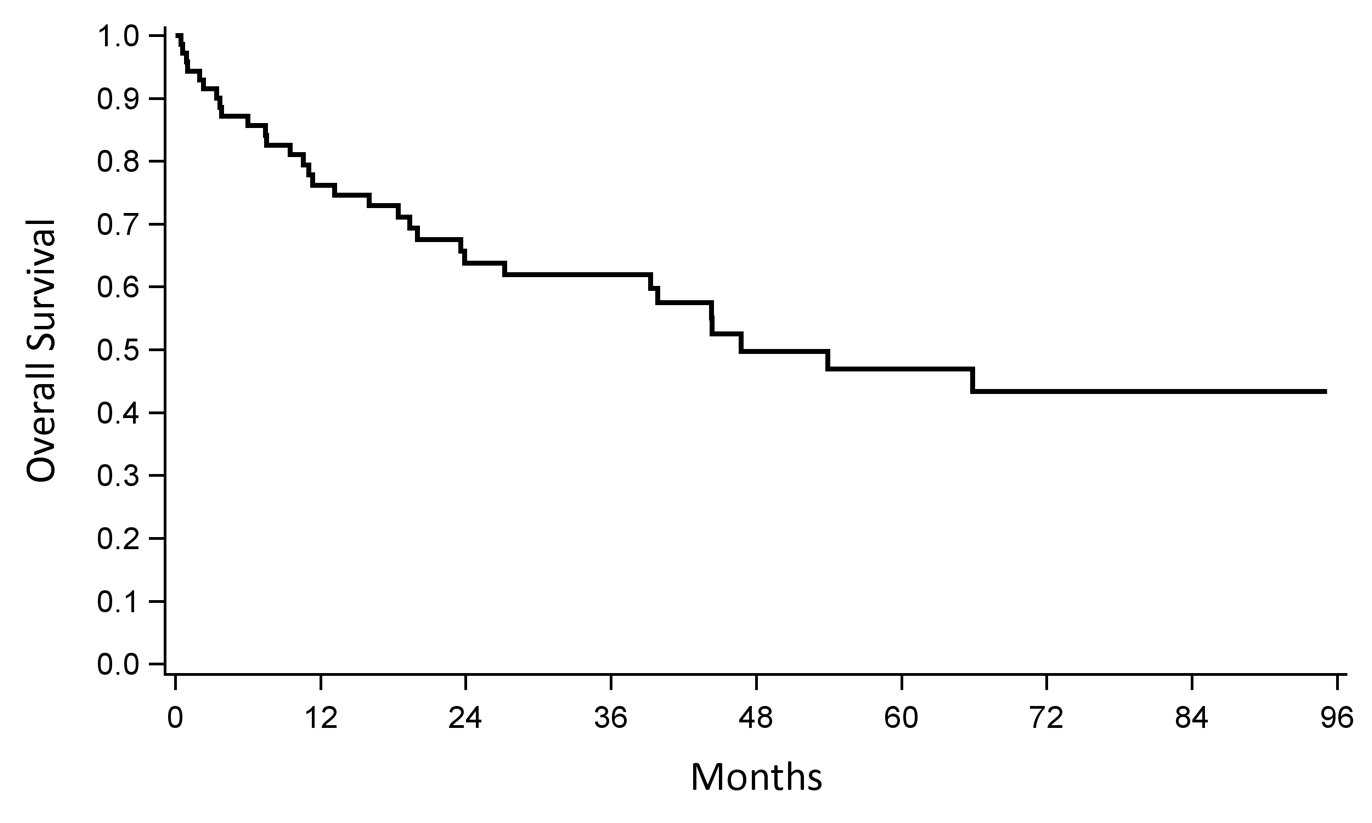


**Figure S2. Overall survival of 140 intermediate-2 and high-risk myelofibrosis patients of Lombardy region after ruxolitinib discontinuation for reasons different from death.**


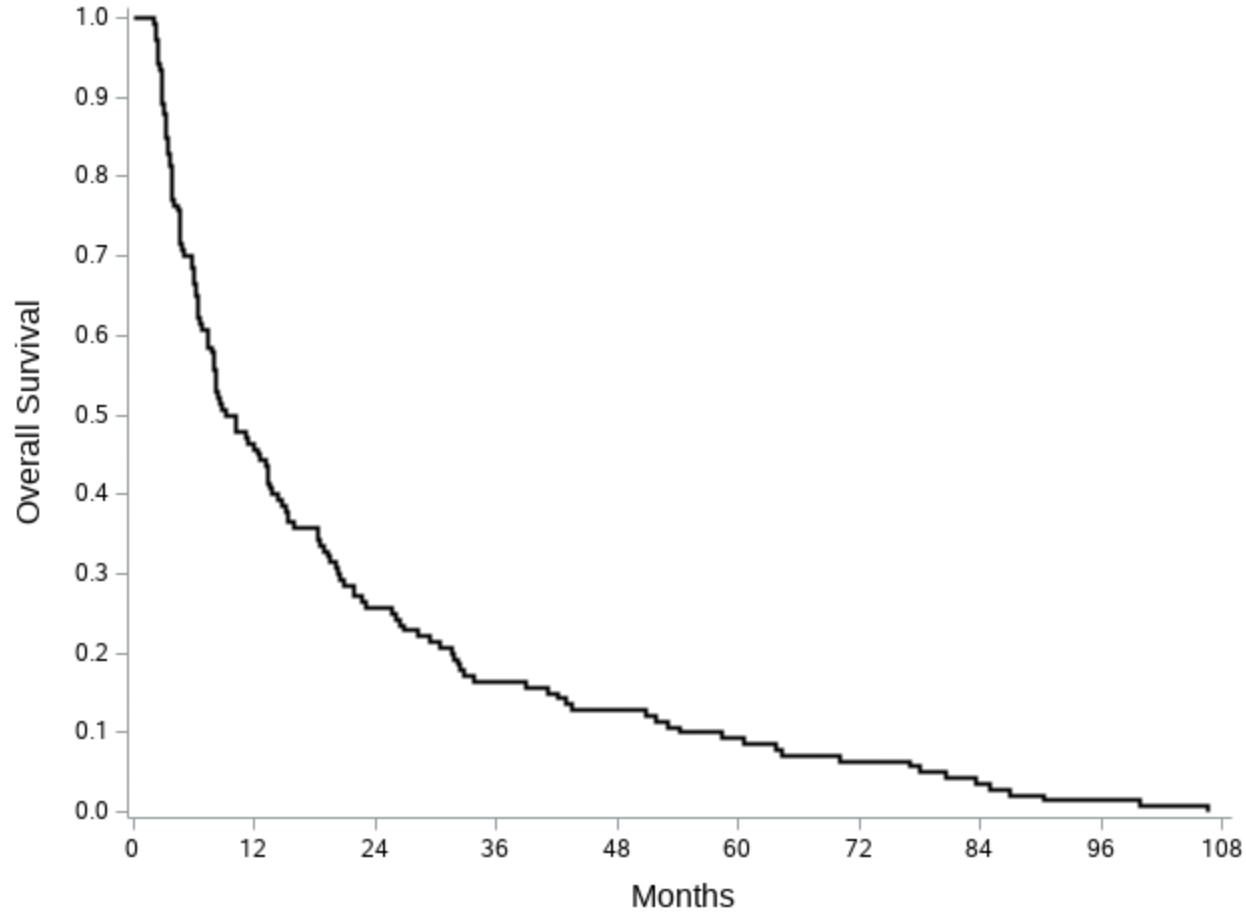

Supplement: Supplementary file 1 — Supporting Information. [file HEM3-10-e70316-s001.docx]
